# Associations between perioperative sleep patterns and clinical outcomes in patients with intracranial tumors: a correlation study

**DOI:** 10.3389/fneur.2023.1242360

**Published:** 2023-09-05

**Authors:** Yang Liu, Fan Wu, Xiaoyu Zhang, Mengyang Jiang, Yiqiang Zhang, Chenhui Wang, Yongxing Sun, Baoguo Wang

**Affiliations:** Department of Anesthesiology, Sanbo Brain Hospital, Capital Medical University, Beijing, China

**Keywords:** perioperative sleep patterns, clinical outcomes, intracranial tumors, sleep disorders, deep sleep

## Abstract

**Objective:**

Although the quality of perioperative sleep is gaining increasing attention in clinical recovery, its impact role remains unknown and may deserve further exploration. This study aimed to investigate the associations between perioperative sleep patterns and clinical outcomes among patients with intracranial tumors.

**Methods:**

A correlation study was conducted in patients with intracranial tumors. Perioperative sleep patterns were assessed using a dedicated sleep monitor for 6 consecutive days. Clinical outcomes were gained through medical records and follow-up. Spearman's correlation coefficient and multiple linear regression analysis were applied to evaluate the associations between perioperative sleep patterns and clinical outcomes.

**Results:**

Of 110 patients, 48 (43.6%) were men, with a median age of 57 years. A total of 618 days of data on perioperative sleep patterns were collected and analyzed. Multiple linear regression models revealed that the preoperative blood glucose was positively related to the preoperative frequency of awakenings (β = 0.125; 95% CI = 0.029–0.221; *P* = 0.011). The level of post-operative nausea and vomiting was negatively related to perioperative deep sleep time (β = −0.015; 95% CI = −0.027–−0.003; *P* = 0.015). The level of anxiety and depression was negatively related to perioperative deep sleep time, respectively (β = −0.048; 95% CI = −0.089–0.008; *P* = 0.020, β = −0.041; 95% CI = −0.076–0.006; *P* = 0.021). The comprehensive complication index was positively related to the perioperative frequency of awakenings (β = 3.075; 95% CI = 1.080–5.070; *P* = 0.003). The post-operative length of stay was negatively related to perioperative deep sleep time (β = −0.067; 95% CI = −0.113–0.021; *P* = 0.005). The Pittsburgh Sleep Quality Index was positively related to perioperative sleep onset latency (β = 0.097; 95% CI = 0.044–0.150; *P* < 0.001) and negatively related to perioperative deep sleep time (β = −0.079; 95% CI = −0.122–0.035; *P* < 0.001).

**Conclusion:**

Perioperative sleep patterns are associated with different clinical outcomes. Poor perioperative sleep quality, especially reduced deep sleep time, has a negative impact on clinical outcomes. Clinicians should, therefore, pay more attention to sleep quality and improve it during the perioperative period.

**Clinical trial registration:**

http://www.chictr.org.cn, identifier: ChiCTR2200059425.

## 1. Introduction

With the development of perioperative medicine and enhanced recovery after surgery, perioperative sleep patterns are becoming major concerns for anesthesiologists and surgeons. Sleep disorders are prevalent in patients during the perioperative period. Previous studies have found sleep disorders in approximately 47.8 to 79.1% of patients before surgery and in 42 to 66.7% of patients after surgery (1–6). Sleep disorders diagnosed perioperatively could persist in plaguing patients for months to years (7, 8). In patients with intracranial tumors, previous studies have found that more than 60% of patients have sleep disorders, with one in five reporting insomnia (9). Perioperative sleep disorders can retard wound healing (10), result in mood disorders and post-operative cognitive dysfunction (7, 11, 12), increase respiratory and cardiovascular complications (13, 14), and prolong hospital stays (2). Different types and degrees of sleep disorders, mainly manifesting as abnormalities in sleep quantity and quality, were under-recognized in clinical studies. Reduced total sleep time, prolonged sleep onset latency, obstructive sleep apnea syndrome, sleep fragmentation, and disrupted sleep architecture are the main perioperative sleep disorders observed in previous studies (15–17). Multiple factors may contribute to or aggravate abnormal sleep during the perioperative period, including factors such as psychological status, ward environment, nursing interventions, surgery, and anesthetic drugs (18).

Different kinds of sleep disorders often lead to varied clinical complications and outcomes due to distinct internal pathophysiological mechanisms. It has been demonstrated that reduced sleep time could activate the microglial cells through microglial voltage-dependent anion channel 1 and increase the activity of L-type calcium channels, leading to increased post-operative pain (19, 20). Obstructive sleep apnea syndrome is deeply associated with post-operative cognitive dysfunction, and the mechanisms involved include neuroinflammation, impairment of hippocampal synaptic plasticity, and blood–brain barrier dysfunction (21–23). Moreover, sleep fragmentation could impair the immune system, alter stress responses, and be associated with perioperative anxiety and depression (24). To the best of our knowledge, the correlation between different perioperative sleep patterns and clinical outcomes is unclear. Moreover, most of the current studies assess perioperative sleep with subjective sleep questionnaires, and prognostic exploration is investigated according to a single index of questionnaire score. Therefore, we carried out a correlation study to investigate the associations between perioperative sleep patterns and clinical outcomes by a dedicated sleep monitor, which could provide multiple sleep parameters reflecting sleep patterns. Our study will lend support to develop accurate and effective sleep treatment strategies for raising the quality of medical care, accelerating post-operative recovery, and improving clinical outcomes for patients.

## 2. Materials and methods

### 2.1. Study design and participants

We conducted a correlation study during a period between January and April 2023 at Sanbo Brain Hospital, Capital Medical University. Patients were eligible for inclusion in the study if they were (1) ≥45 years, (2) American Society of Anesthesiologists physical status classification ≤ II, (3) undergoing elective craniotomy for intracranial tumors, and (4) without dissemination and distant metastasis. Patients were excluded if they were (1) unable to complete assessments due to various reasons or (2) participating in an intervention study related to this study. All potential participants provided written informed consent. The study was approved by the institutional ethics committee (SBNK-YJ-2022-011-01) and registered on the Chinese Clinical Trial Registry (ChiCTR2200059425).

### 2.2. Assessment of perioperative sleep patterns

**Figure 1:**
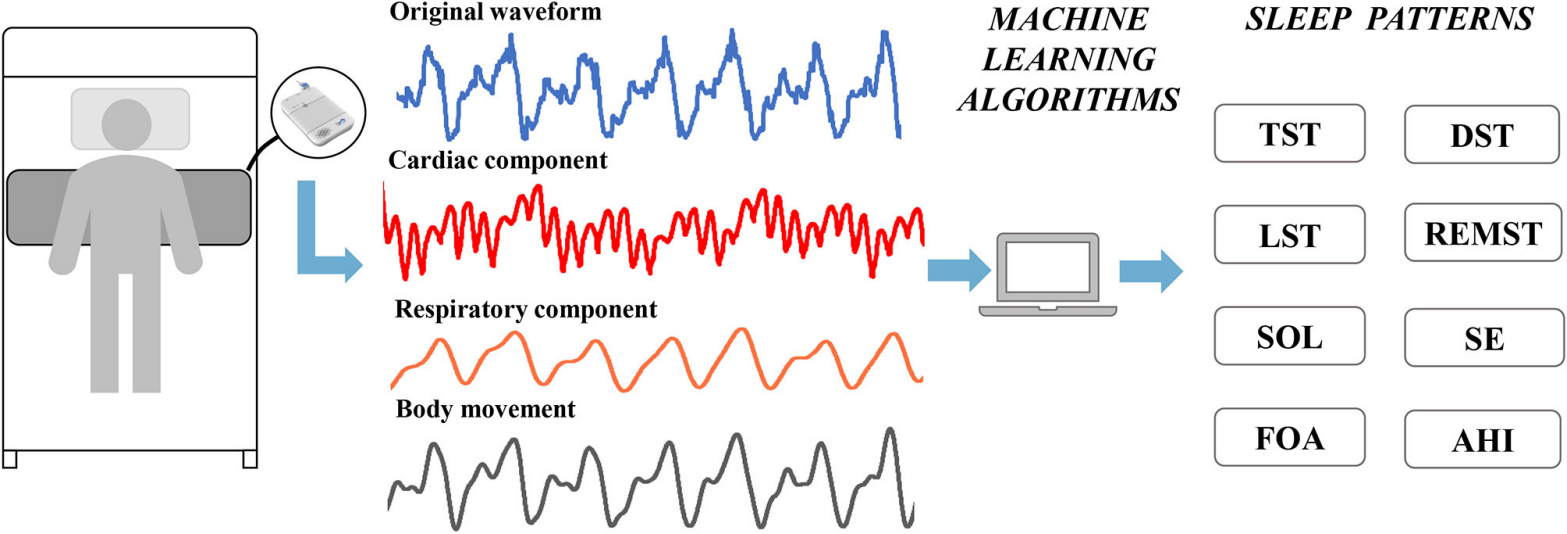
Applications of sleep monitors to perioperative sleep patterns estimation. TST, total sleep time; DST, deep sleep time; LST, light sleep time; REMST, rapid eye movement sleep time; SOL, sleep onset latency; SE, sleep efficiency; FOA, frequency of awakenings; AHI, apnea–hypopnea index.

Perioperative sleep patterns were collected by a dedicated sleep monitor (SC-500^TM^; Boshi Linkage Technology, Beijing, China) with a built-in electret condenser microphone (EM246ASS^TM^; Hakujitsu Technology Co., Tokyo, Japan) (Figure 1). The sleep monitor can detect 0.01–10 kHz frequency domain signals, enabling accurate separation of vital sign information such as heart rate (0.8–1.5 Hz) and respiration (0.2–0.8 Hz). The perioperative sleep pattern is based on the algorithm of ballistocardiogram and consists of two main parts (25). The first part is the sleep stage, including total sleep time (TST), deep sleep time (DST), light sleep time (LST), and rapid eye movement sleep time (REMST), and the second part is the sleep characteristics, including sleep onset latency (SOL), sleep efficiency (SE), frequency of awakenings (FOA), and apnea–hypopnea index (AHI). Previous studies have proven the accuracy of this sleep monitor, using polysomnography as the gold standard (26, 27). We performed 6 consecutive days of sleep pattern monitoring 3 days before and 3 days after surgery.

### 2.3. Management of anesthesia

Electrocardiogram, oxygen saturation, invasive arterial blood pressure, body temperature, and entropy index were routinely monitored before surgery. Heart rate, blood pressure, and blood glucose were recorded immediately after admission to the operating room.

Anesthesia was induced with a standardized regimen of propofol, sufentanil, and rocuronium. The anesthesia maintenance drugs that were used included propofol, rocuronium, remifentanil, and sufentanil. The entropy index was maintained between 40 and 60 during surgery. Anesthesiologists maintained mean arterial blood pressure within 20% of baseline measurements by adjusting the depth of anesthesia and vasoactive drug dosage. All patients received 48 h of post-operative analgesic management, and analgesic pump medications were standardized to dezocine (0.6 mg/kg) and tropisetron 10 mg, configured into 100 mL of saline. At the end of the surgery, the patient-controlled analgesic pump was used for continuous administration of 2 ml/h and self-administration of 4 ml/dose, with an interlock time of 15 min. Anesthesia-related data were obtained from the anesthetic records.

### 2.4. Comprehensive complication index

The comprehensive complication index (CCI) is a new method for evaluating all post-operative complications (28). The index is calculated according to the Clavien–Dindo classification of every complication and its severity weighting and provides a total score for each patient from 0 (no complications) to 100 (death).

We recorded all complications from the first to the seventh post-operative day by follow-up and graded the complications according to the Clavien–Dindo classification and calculated the CCI.

### 2.5. Demographic and clinical characteristics collection

Demographic information (gender, age, body mass index, years of education, drinking, smoking, and medical history) was collected at admission. Subjective sleep quality (Pittsburgh Sleep Quality Index, PSQI), cognitive function (Mini-Mental State Examination), and the activity of daily living (Barthel Index) were assessed on the day of admission and 30 days post-operatively by follow-up. The degree of pain, nausea, and vomiting and symptoms of anxiety and depression were evaluated using the Numerical Rating Scale (NRS) and the Hospital Anxiety and Depression Scale (HADS) on the day of admission and the 7th post-operative day. The results of laboratory tests (routine blood tests and blood biochemistry tests) conducted at the admission and the first 24 h after surgery were collected. Information on length of stay and costs was collected through the electronic medical records.

### 2.6. Statistical analysis

The normality of the variable's distribution was analyzed using the Kolmogorov–Smirnov test. The data were presented as mean ± standard deviation (SD), median (interquartile range, IQR), and frequency (percentage), as appropriate.

The points of the perioperative sleep trend are depicted as mean values. Spearman's correlation coefficient was used to evaluate the correlation between perioperative sleep patterns and clinical outcomes. A multiple linear regression analysis with a stepwise method was used to examine the effect of different perioperative sleep pattern parameters on clinical outcomes. The parameters were selected using Spearman's correlation coefficient (*P*-value < 0.05) and adjusted by age, gender, and BMI. In all models, we have also evaluated any potential influence of multicollinearity, independency, and residual variance homogeneity to ensure the accuracy of the model. A *P*-value of < 0.05 was considered to be statistically significant in all analyses. All statistical analyses were performed using SPSS Statistics 25.0 (IBM Corp, Armonk, NY, USA).

## 3. Results

### 3.1. Characteristics of study participants

A total of 110 participants were included in this study (Table 1). The median age of all participants was 57 years [interquartile range (IQR), 50–63 years], of which 43.6% were men. A history of sleep medication administration was present in 6.4% of patients. The median PSQI of all patients on the day of admission was 6, with an interquartile range of 3–10.

**Table 1:** Demographic and clinical characteristics of 110 participants.

**Characteristics (*N* = 110)**	**Value**
Age, years	57 (50–63)
Gender, Male/Female	48/62
BMI, kg/m^2^	24.69 ± 3.62
Years of education, years	9.0 (6.0–12.0)
Drinking, *n* (%)	19 (17.3)
Smoking, *n* (%)	23 (20.9)
Hypertension, *n* (%)	29 (26.4)
Diabetes, n (%)	11 (10.0)
Sleep medications, *n* (%)	7 (6.4)
Antipsychotic medications, *n* (%)	4 (3.6)
Hemoglobin, g/L	136.15 ± 13.10
Albumin, g/L	39.22 ± 3.01
Glucose, mmol/L	5.2 (4.8–5.9)
**Tumor classification**, ***n*** **(%)**	
Meningioma	43 (39.1)
Glioma	42 (38.2)
Acoustic neuroma	11 (10.0)
Other	14 (12.7)
**Tumor location**, ***n*** **(%)**	
Supratentorial	79 (71.8)
Infratentorial	31 (28.2)
Malignant, *n* (%)	52 (47.3)
Tumor size, cm^3^	43.5 (17.9–95.9)
Barthel index	90 (80–95)
PSQI	6 (3–10)
MMSE	27 (25–29)
HADS -anxiety score	3 (0–5)
HADS -depression score	3 (0–5)

BMI, body mass index; PSQI, Pittsburgh Sleep Quality Index; MMSE, Mini-Mental State Examination; HADS, Hospital Anxiety and Depression Scale. Data are presented as mean ± standard deviation, median (interquartile range), or frequency (percentage) as appropriate.

### 3.2. The trend of perioperative sleep patterns

**Figure 2:**
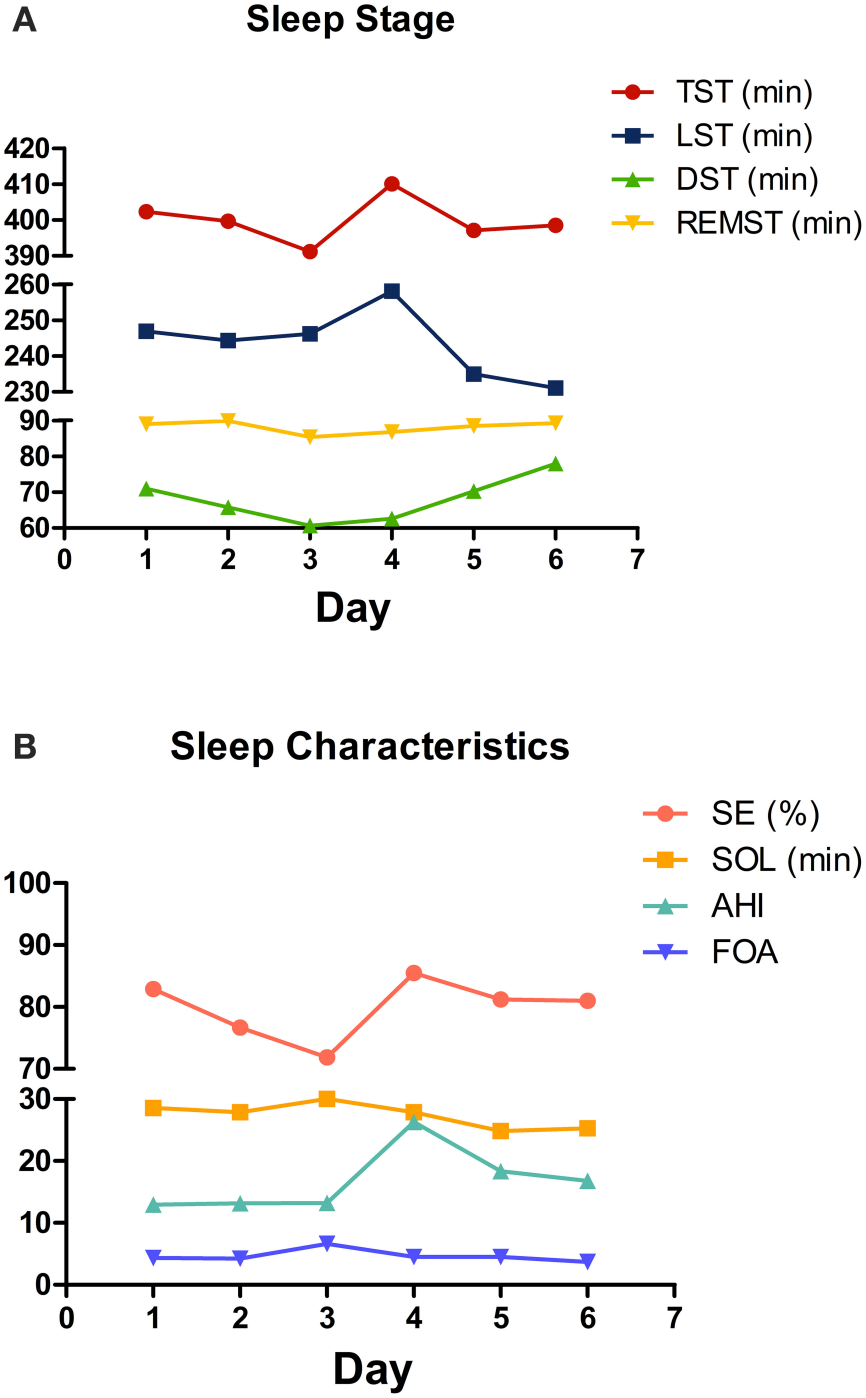
Changing trend of perioperative sleep patterns. **(A)** Sleep stage; **(B)** sleep characteristics. The mean values are plotted for each sleep pattern for each day.

A total of 618 days of data on perioperative sleep patterns were collected and described in Figure 2. Regarding the sleep stage, the TST and DST showed a gradual decrease during 3 days before surgery, while LST was relatively stable. In the post-operative phase, TST and LST were significantly higher on the first post-operative day and gradually decreased thereafter. The DST gradually increased in the post-operative period, and the time of REM sleep was relatively stable throughout the perioperative period (Figure 2A). Regarding sleep characteristics, SE was in a gradual decrease in the preoperative phase, with a significant increase on the first post-operative day, followed by a gradual decrease. The AHI was significantly higher on the first post-operative day. SOL and FOA were in a relatively stable trend throughout the perioperative period (Figure 2B).

### 3.3. Pre-operative sleep patterns and the outcome on the day of surgery

We analyzed the correlation between the mean values of pre-operative 3-day sleep patterns and vital signs immediately after entering the operating room, the dosage of the sedative and analgesic drugs, and the inflammatory indicators conducted within the first 24 h after surgery (Table 2).

**Table 2:** Associations between preoperative sleep patterns and the outcome on the day of surgery.

		**TST-pre**	**DST-pre**	**LST-pre**	**REMST-pre**	**SOL-pre**	**SE-pre**	**FOA-pre**	**AHI-pre**
**Perioperative outcomes**									
Heart rate	*r*	0.142	0.146	−0.019	0.040	−0.141	0.113	−0.038	0.095
	*p*	0.139	0.127	0.844	0.680	0.141	0.240	0.696	0.326
Systolic pressure, mmHg	*r*	−0.058	−0.086	−0.091	0.152	−0.029	0.118	−0.081	0.095
	*p*	0.548	0.371	0.343	0.114	0.762	0.221	0.403	0.322
Diastolic pressure, mmHg	*r*	0.014	−0.042	−0.059	0.082	0.056	0.062	0.035	0.180
	*p*	0.887	0.661	0.538	0.396	0.564	0.522	0.714	0.060
Glucose, mmol/L	*r*	−0.190	−0.248	−0.098	−0.021	0.100	−0.071	0.234	−0.023
	*p*	**0.047**	**0.009**	0.310	0.831	0.300	0.462	**0.014**	0.815
Dosage of propofol, mg	*r*	−0.015	0.033	−0.108	0.127	0.058	0.007	−0.055	0.082
	*p*	0.876	0.734	0.263	0.190	0.552	0.944	0.573	0.398
**Dosage of opioids, ug**									
Sufentanil	*r*	−0.092	0.036	−0.172	−0.022	0.171	0.006	−0.134	0.101
	*p*	0.519	0.799	0.223	0.877	0.226	0.965	0.343	0.475
Remifentanil	*r*	0.117	0.066	0.172	−0.032	−0.035	0.041	0.016	0.099
	*p*	0.394	0.631	0.209	0.818	0.802	0.768	0.905	0.470
Dosage of vasoactive drugs, mg	*r*	−0.069	−0.120	0.106	0.017	0.159	−0.126	0.068	−0.033
	*p*	0.472	0.213	0.270	0.860	0.097	0.188	0.481	0.733
Neutrophil-to-lymphocyte ratio	*r*	−0.192	−0.053	−0.205	−0.108	0.090	−0.078	0.219	0.232
	*p*	**0.044**	0.581	**0.032**	0.262	0.351	0.421	**0.021**	**0.015**
Monocyte-to-lymphocyte ratio	*r*	0.029	0.019	0.045	−0.154	−0.100	0.114	0.046	−0.110
	*p*	0.762	0.847	0.641	0.109	0.297	0.236	0.631	0.251
C-reactive protein, mg/L	*r*	0.007	−0.076	0.089	0.014	−0.099	0.036	−0.002	−0.084
	*p*	0.939	0.432	0.354	0.886	0.303	0.711	0.987	0.381

TST-pre, preoperative total sleep time; DST-pre, preoperative deep sleep time; LST-pre, preoperative light sleep time; REMST-pre, preoperative rapid eye movement sleep time; SOL-pre, preoperative sleep onset latency; SE-pre, preoperative sleep efficiency; FOA-pre, preoperative frequency of awakenings; AHI-pre, preoperative apnea-hypopnea index. The bold values indicate the *p*-value of < 0.05.

Correlation analyses revealed negative associations between the level of blood glucose and TST (*r*_*s*_ = −0.190, *P* = 0.047) and DST (*r*_*s*_ = −0.248, *P* = 0.009), respectively. A positive association was found between the level of glucose and FOA (*r*_*s*_ = 0.234, *P* = 0.014). In addition, negative associations were found between neutrophil-to-lymphocyte ratio and TST (*r*_*s*_ = −0.192, *P* = 0.044) and LST (*r*_*s*_ = −0.205, *P* = 0.032), respectively. Positive associations were found between neutrophil-to-lymphocyte ratio and FOA (*r*_*s*_ = −0.219, *P* = 0.021) and AHI (*r*_*s*_ = −0.232, *P* = 0.015), respectively.

**Figure 3:**
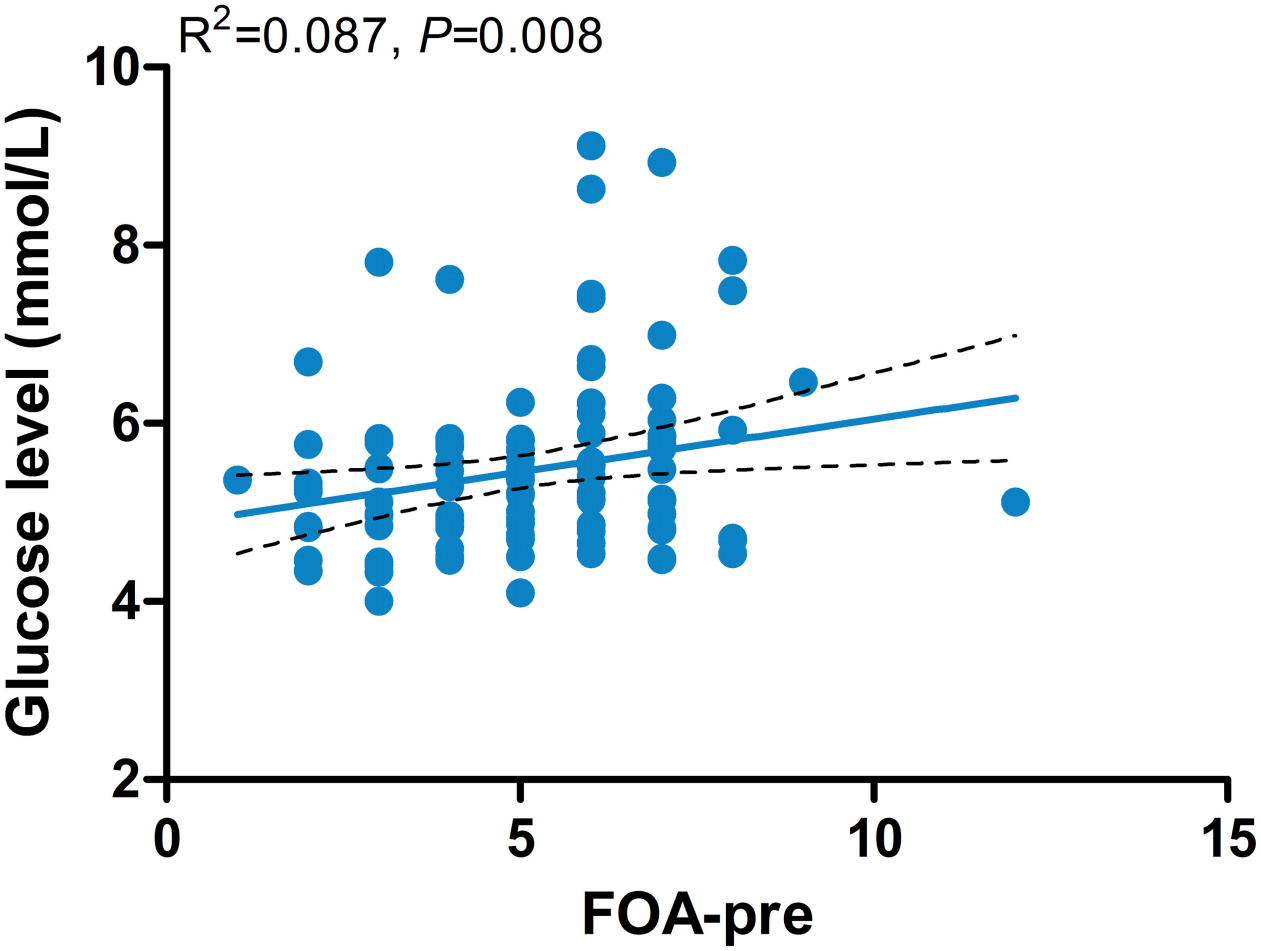
Associations between the level of blood glucose and FOA-pre. FOA-pre, preoperative frequency of awakenings.

The results of the multiple linear regression models showed that the level of blood glucose was positively related to FOA (β = 0.125; 95% CI = 0.029–0.221; *P* = 0.011) (Figure 3). No sleep patterns were related to the neutrophil-to-lymphocyte ratio.

### 3.4. Perioperative sleep patterns and post-operative outcomes

We analyzed the correlation between the mean values of perioperative 6-day sleep patterns and post-operative outcomes (Table 3). Correlation analyses revealed negative associations between NRS-post-operative nausea and vomiting (PONV) and DST (*r*_*s*_ = −0.245, *P* = 0.010). Negative associations between the HADS-anxiety score and TST (*r*_*s*_ = −0.225, *P* = 0.018), and DST (*r*_*s*_ = −0.280, *P* = 0.003), respectively, were found as well. In addition, the HADS-depression score was negatively associated with TST (*r*_*s*_ = −0.289, *P* = 0.002), DST (*r*_*s*_ = −0.293, *P* = 0.002), and SE (*r*_*s*_ = −0.215, *P* = 0.024) and positively associated with SOL (*r*_*s*_ = 0.317, *P* = 0.001) and FOA (*r*_*s*_ = 0.209, *P* = 0.028). CCI was positively associated with SOL (*r*_*s*_ = 0.195, *P* = 0.042) and FOA (*r*_*s*_ = 0.235, *P* = 0.013), respectively. Furthermore, post-operative length of stay was negatively associated with DST (*r*_*s*_ = −0.213, *P* = 0.025) and positively associated with AHI (*r*_*s*_ = 0.224, *P* = 0.019). Finally, the PSQI-3 month was negatively associated with TST (*r*_*s*_ = −0.487, *P* < 0.001), DST (*r*_*s*_ = −0.479, *P* < 0.001), REMST (*r*_*s*_ = −0.338, *P* < 0.001), and SE (*r*_*s*_ = −0.496, *P* < 0.001) and positively associated with SOL (*r*_*s*_ = 0.572, *P* < 0.001), FOA (*r*_*s*_ = 0.449, *P* < 0.001), and AHI (*r*_*s*_ = 0.266, *P* = 0.005).

**Table 3:** Associations between perioperative sleep patterns and post-operative outcomes.

		**TST-peri**	**DST-peri**	**LST-peri**	**REMST-peri**	**SOL-peri**	**SE-peri**	**FOA-peri**	**AHI-peri**
**Postoperative outcomes**									
NRS-pain	*r*	0.107	0.047	0.078	0.029	−0.033	−0.012	0.076	0.040
	*p*	0.264	0.625	0.420	0.762	0.734	0.898	0.432	0.680
NRS-PONV	*r*	−0.085	−0.245	0.152	0.001	0.055	−0.045	0.069	0.150
	*p*	0.379	**0.010**	0.114	0.992	0.566	0.642	0.473	0.117
HADS-anxiety score	*r*	−0.225	−0.280	−0.028	−0.105	0.162	−0.110	0.118	0.093
	*p*	**0.018**	**0.003**	0.772	0.274	0.091	0.251	0.218	0.332
HADS-depression score	*r*	−0.289	−0.293	−0.055	−0.182	0.317	−0.215	0.209	0.147
	*p*	**0.002**	**0.002**	0.570	0.057	**0.001**	**0.024**	**0.028**	0.125
Days of wound healing, days	*r*	0.098	0.013	0.113	0.143	0.000	−0.073	−0.031	0.139
	*p*	0.310	0.894	0.239	0.136	0.996	0.449	0.752	0.147
Comprehensive complication index	*r*	0.003	−0.042	0.029	−0.079	0.195	−0.111	0.235	0.073
	*p*	0.971	0.666	0.762	0.410	**0.042**	0.248	**0.013**	0.450
Length of stay, days	*r*	−0.008	−0.135	−0.049	0.158	0.088	0.052	−0.036	0.146
	*p*	0.936	0.159	0.608	0.099	0.361	0.589	0.706	0.128
Postoperative length of stay, days	*r*	−0.078	−0.213	0.051	0.061	0.079	0.036	0.052	0.224
	*p*	0.419	**0.025**	0.599	0.524	0.411	0.709	0.589	**0.019**
Hospitalization costs, RMB	*r*	0.04	−0.11	0.027	0.099	0.136	−0.058	0.002	0.165
	*p*	0.676	0.253	0.78	0.304	0.156	0.545	0.987	0.085
Barthel index-3 month	*r*	0.145	0.066	0.165	0.092	−0.105	0.074	−0.095	−0.161
	*p*	0.130	0.491	0.085	0.339	0.275	0.442	0.322	0.094
PSQI-3 month	*r*	−0.487	−0.479	−0.056	−0.338	0.572	−0.496	0.449	0.266
	*p*	**< 0.001**	**< 0.001**	0.562	**< 0.001**	**< 0.001**	**< 0.001**	**< 0.001**	**0.005**
MMSE-3 month	*r*	0.019	0.096	0.029	−0.031	−0.008	−0.082	−0.015	−0.052
	*p*	0.844	0.319	0.762	0.747	0.934	0.396	0.88	0.59

TST-peri, perioperative total sleep time; DST-peri, perioperative deep sleep time; LST-peri, perioperative light sleep time; REMST-peri, perioperative rapid eye movement sleep time; SOL-peri, perioperative sleep onset latency; SE-peri, perioperative sleep efficiency; FOA-peri, perioperative frequency of awakenings; AHI-peri, perioperative apnea-hypopnea index. NRS, Numerical Rating Scale; PONV, post-operative nausea and vomiting; HADS, Hospital Anxiety and Depression Scale; PSQI, Pittsburgh Sleep Quality Index; MMSE, Mini-Mental State Examination. The bold values indicate the value of *p* < 0.05.

**Figure 4:**
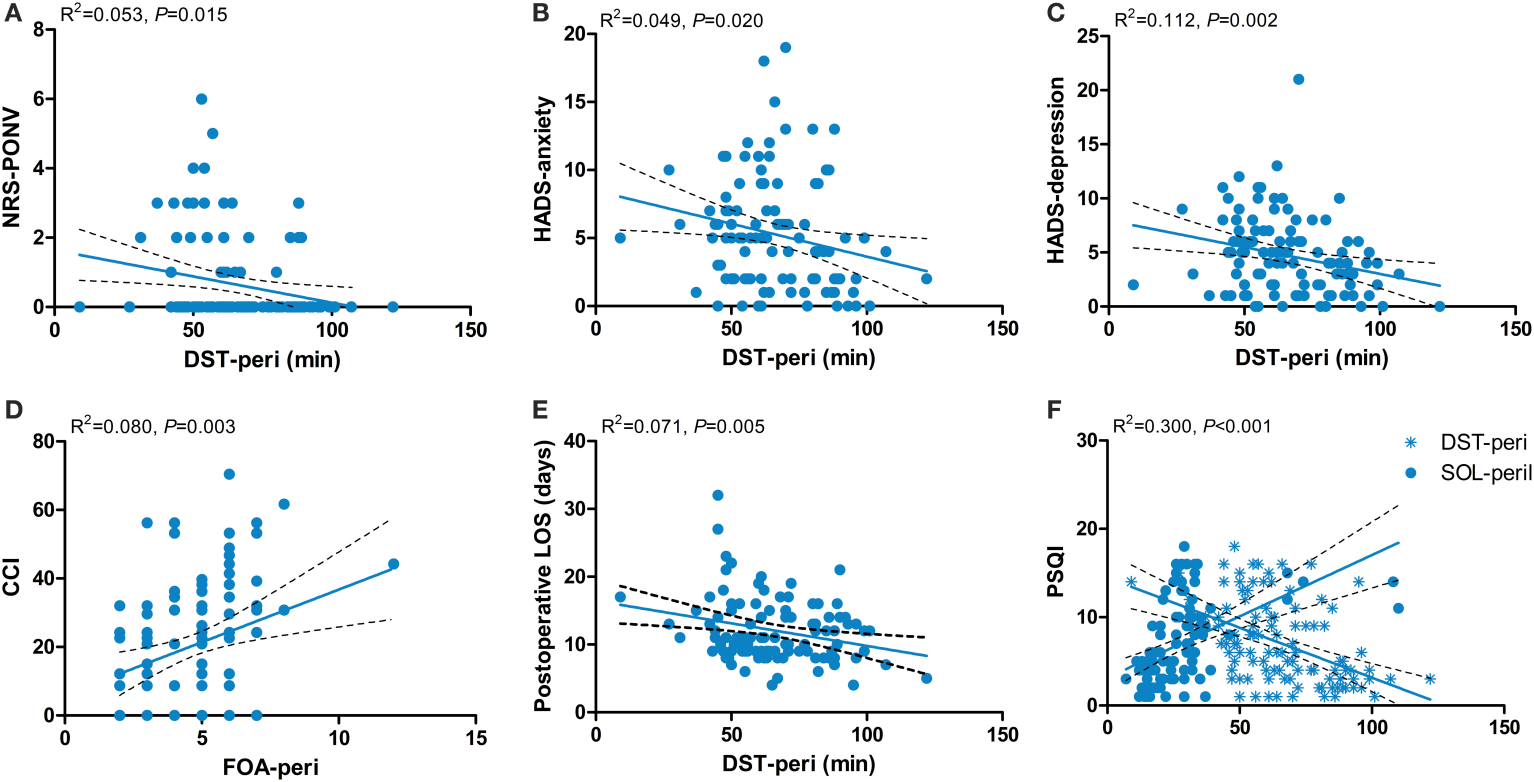
Associations between perioperative sleep patterns and post-operative outcomes. **(A)** NRS-PONV; **(B)** HADS-anxiety; **(C)** HADS-depression; **(D)** CCI; **(E)** Postoperative LOS; **(F)** PSQI-3 month. NRS, Numerical Rating Scale; PONV, post-operative nausea and vomiting; HADS, Hospital Anxiety and Depression Scale; CCI, Comprehensive Complication Index; LOS, length of stay; PSQI, Pittsburgh Sleep Quality Index; DST-peri, perioperative deep sleep time; FOA-peri, perioperative frequency of awakenings; SOL-peri, perioperative sleep onset latency.

The results of the multiple linear regression models showed that the NRS-PONV was negatively related to DST (β = −0.015; 95% CI = −0.027–−0.003; *P* = 0.015) (Figure 4A). The HADS-anxiety score was negatively related to DST (β = −0.048; 95% CI = −0.089–−0.008; *P* = 0.020) (Figure 4B). The HADS-depression score was negatively related to DST (β = −0.041; 95% CI = −0.076–−0.006; *P* = 0.021) (Figure 4C). The CCI was positively related to FOA (β = 3.075; 95% CI = 1.080–5.070; *P* = 0.003) (Figure 4D). The post-operative length of stay was negatively related to DST (β = −0.067; 95% CI = −0.113–−0.021; *P* = 0.005) (Figure 4E). The PSQI-3 month was positively related with SOL (β = 0.097; 95% CI = 0.044–0.150; *P* < 0.001) and negatively related with DST (β = −0.079; 95% CI = −0.122–−0.035; *P* < 0.001) (Figure 4F).

## 4. Discussion

We investigated 110 patients with intracranial tumors who received 6 days of perioperative sleep monitoring to carry out a correlation analysis between perioperative sleep patterns and clinical outcomes. To the best of our knowledge, our study demonstrated, for the first time, the changing trend of different sleep patterns during the perioperative period. Moreover, we found that the level of preoperative blood glucose is positively related to preoperative FOA. The level of PONV, post-operative anxiety and depression, post-operative length of stay, and the PSQI scores assessed 3 months after surgery is negatively related to the perioperative DST. The CCI is positively related to the perioperative FOA. The PSQI score assessed 3 months after surgery is positively related to the perioperative SOL.

The changing trend of perioperative sleep patterns is not yet known. In recent studies, sleep patterns were monitored on separate days during the perioperative period using polysomnography or other sleep-monitoring devices. In this study, the sleep patterns were recorded continuously by a dedicated sleep monitor for 3 days before and 3 days after surgery, reflecting the perioperative sleep quality of patients from a multidimensional perspective. The changing trend of TST, DST, and SE is consistent with the results of previous studies and may be due to negative emotions and changes in the sleep environment (29). Negative emotions such as tension, anxiety, and depression are usually observed in patients before surgery. Some studies have identified that anxiety can cause reduced sleep time through the dysregulation of neurotransmitter systems such as cholinergic and Gamma-aminobutyric acid (GABA) (30). Moreover, sleep disorders are the major symptoms of depression, manifested by decreased sleep efficiency and increased REM sleep time (31). The relationship is likely to be bidirectional as sleep disorders worsen negative emotions, while negative emotions similarly exacerbate sleep disorders (18). Hospital environmental factors can also compromise sleep quality. One study showed that new-onset insomnia was reported by 36% of hospitalized patients, with 10% of these being clinical and severe insomnia (32). In the post-operative phase, due to the effects of the anesthesia and the surgery, the patient's sleep structure is greatly altered. The type and site of surgery have a significant impact on perioperative sleep patterns. Studies have confirmed that the incidence of sleep disorders and altered polysomnograms is more pronounced after major surgery than after minor surgery (33). Surgery-induced inflammatory factors such as interleukin (IL)-1β, IL-6, and tumor necrosis factor-α are important factors in the regulation of sleep architecture (34). Moreover, patients who underwent emergency surgery were 2.46 times more likely to develop sleep disorders compared to those who underwent elective surgery (6). The effect of different anesthesia methods on sleep patterns varies. General anesthesia induces clock disruption by affecting major neurotransmitter systems, including GABA/NMDA, which in turn impairs perioperative sleep patterns (35, 36). Bellet et al. (37) found that ketamine could alter the recruitment of the CLOCK: BMAL1 complex, leading to circadian dysfunction. Compared to general anesthesia, patients with local anesthesia have better sleep quality at night. This may be because local anesthesia requires fewer anesthetic drugs (especially opioids) (6, 17). The rebound in the REMST is a typical phenomenon during the perioperative period (38). Due to opioid administration, however, we did not find this phenomenon. We assume that this may be explained by the fact that we monitored up to the third post-operative day, but this occurred mostly on the 3rd to 5th post-operative day. Regarding sleep characteristics, the AHI was significantly higher on the first post-operative day. First, patients with craniocerebral tumors are prone to upper airway obstruction due to the weakened support and control of the airway by the brain center post-operatively, and second, the immediate post-operative supine position leads to a decrease in lung volume and oxygen saturation. In addition, previous studies have shown that REM sleep is associated with a loss of pharyngeal muscle tone, leading to upper respiratory tract obstruction, which increases the likelihood of obstructive sleep apnea syndrome (38, 39), but this correlation was not found in our study.

Sleep is an important physiological function. It allows our body to rest and speed up recovery. Previous studies have demonstrated that perioperative sleep disorders may increase the body's stress response, lead to greater hemodynamic changes, and affect the emotional state and ability to heal (40). However, preoperative sleep quality has not received sufficient attention in clinical studies. In our study, we found that the preoperative blood glucose level is positively related to the preoperative FOA. Our result was consistent with previous studies, which have confirmed that blood glucose levels are to some extent related to the stress response of the body, and sleep fragmentation could increase stress and damage glucose metabolism (41, 42). The higher the number of preoperative awakenings, the more stressful the patient is, which not only increases the risk of surgery but also poses a challenge for post-operative recovery, especially wound healing.

A healthy perioperative sleep pattern is characterized by both quantity and quality. The association between perioperative sleep patterns and post-operative outcomes has been explored in several studies. However, our study did not grossly dichotomize sleep disorders in a qualitative way to explore their association with clinical outcomes. This is because different types of sleep disorders may trigger different clinical outcomes through their corresponding mechanisms. Therefore, exploring that association is of great importance for the development of accurate and effective sleep interventions and treatment plans. In this study, we found that perioperative DST was related to several post-operative clinical outcomes, including PONV, post-operative anxiety and depression, post-operative length of stay, and PSQI scores at 3 months post-operatively. Deep sleep accounts for 15–25% of nocturnal sleep time and serves as an important component of sleep. Deep sleep has been demonstrated as a major factor in energy saving, hormone release, regulating the immune system and metabolism, and consolidating cognitive functions (43). First, we found that the shorter the duration of deep sleep, the more severe the PONV. The result is consistent with a longitudinal study conducted in 196 cancer patients (44). However, in a meta-analysis of 416 patients with total hip or knee arthroplasty, the authors found that increased DST did not improve PONV. This is not consistent with our results because we focused on the severity of PONV, whereas the previous study focused more on its incidence (45). Second, we found that reduced DST could aggravate anxiety and depression during the perioperative period. The role of sleep in the regulation of emotion cannot be ignored. Sufficient sleep time assists in regulating the balance of neurotransmitters such as dopamine, glutamate, serotonin, and adenosine to prevent anxiety (46). Furthermore, sleep deprivation has been demonstrated to further contribute to the onset and progression of depression through activation of the sympathetic nervous system and β-adrenergic signaling, which increased the level of inflammatory markers such as IL-6 and C-reactive protein (47). In some randomized controlled studies, perioperative anxiety and depression were significantly alleviated by the administration of sleep-improving drugs such as melatonin or zolpidem (12, 48). Third, the results demonstrated a correlation between the time of deep sleep and the length of hospitalization. We speculate that this may be related to the regulatory effect of deep sleep on the immune system. Previous studies found that deep sleep regulates inflammatory factors and multiple immune cells including natural killer cells, antigen-presenting cells, and T helper cells, thereby reducing the incidence of inflammation-related complications (49). Generally, a reduction in post-operative complications can shorten the length of post-operative hospital stay. Fourth, sleep disorders that occur during the perioperative period may last for months or even years (50). Results showed that shorter perioperative DST and longer SOL were associated with poorer sleep quality at 3 months post-operatively, as manifested by higher PSQI scores. Our finding also further confirms that perioperative sleep problems will continue to disturb patients for a certain period and affect sleep quality in the long term. Fifth, the CCI is positively related to the perioperative FOA. The CCI is the most widely used index for grading post-operative complications in most surgical areas. All post-operative complications classified according to the Clavien–Dindo classification in this study can be seen in Supplementary Table 1. The reason for choosing this index over a single post-operative complication in this study was to better investigate the impact of perioperative sleep patterns on clinical outcomes in a comprehensive manner. A higher frequency of awakenings indicates a more pronounced degree of sleep fragmentation. Xie et al. have proven that sleep fragmentation induces endosomal-autophagosome-lysosomal pathway dysfunction and microglia-mediated neuroinflammation (24). In addition, sleep fragmentation can lead to blood–brain barrier disruption and neuronal damage (51). These pathological changes make patients more susceptible to neurological complications such as post-operative cognitive dysfunction. An increased risk of cardiovascular complications (e.g., stroke, myocardial infarction, and atrial fibrillation) is associated with sleep fragmentation as well (52). Moreover, the effects of sleep fragmentation on the corticotropic axis also make patients more susceptible to hormonal and electrolyte disturbances (53). Therefore, more severe sleep fragmentation may lead to higher post-operative complication scores. Lastly, increased post-operative pain levels and impaired cognitive function are the two most common adverse outcomes caused by perioperative sleep disorders (54). However, no relevant evidence was found in this study. Concerning post-operative pain, this may be because our patients all had adequate multimodal post-operative analgesia after surgery. The level of post-operative pain was mild during the post-operative follow-up. In addition, we followed up on cognitive function in the post-operative period but ultimately in the form of CCI for the analysis. In conclusion, poor perioperative sleep patterns have a negative impact on clinical outcomes. Therefore, clinicians should pay more attention to perioperative sleep of patients. The targeted perioperative screening and monitoring specifically for sleep patterns are sorely required to improve the short- and long-term outcomes of patients.

There are some limitations in the present study. First, our study only included patients with intracranial tumors, so the results may be limited to extrapolation to other populations. Craniotomy, as a relatively large operation, has a long post-operative recovery time and more complications, which is conducive to the study of the relationship between perioperative sleep patterns and clinical outcomes. In addition, the relative importance of optimizing sleep before and after large surgery may be greater than that of minor surgery, which has less impact on the clinical outcomes. Second, the sleep patterns used for the correlation analysis of our study were obtained from a dedicated sleep monitor and not from gold-standard polysomnography. While the sleep monitor is significantly more accessible for sleep assessment and has a high correlation with polysomnography, it may still need to be more accurate.

## 5. Conclusion

In conclusion, our study first describes the changing trend of perioperative sleep patterns in patients with intracranial tumors. In addition, our study provides evidence for the associations between perioperative sleep patterns and different clinical outcomes. The high prevalence of perioperative sleep disorders and the detrimental effects on clinical outcomes help to make a more in-depth study of perioperative sleep of significant value. Further studies should investigate the underlying mechanisms linking perioperative sleep and clinical outcomes to provide a more targeted clinical guidance.

## Data availability statement

The raw data supporting the conclusions of this article will be made available by the authors, without undue reservation.

## Ethics statement

The studies involving human participants were reviewed and approved by the Research Ethics Committee of Sanbo Brain Hospital of Capital Medical University (SBNKYJ-2022-011-01). The patients/participants provided their written informed consent for participation.

## Author contributions

YL, YS, and BW: study concept and design. FW, MJ, YZ, and CW: data collection. YL and XZ: statistical analysis. YL and FW: drafting of the manuscript. BW: funding. YS and BW: study supervision. All authors contributed significantly to the manuscript.
