# Supplementary material for: Associations between perioperative sleep patterns and clinical outcomes in patients with intracranial tumors: a correlation study
Source: Front Neurol. 2023 Sep 5;14:1242360. doi: 10.3389/fneur.2023.1242360 (PMC10508910; doi:10.3389/fneur.2023.1242360)
Supplement: Supplementary file 1 [file Table_1.pdf]

Associations between perioperative sleep patterns and clinical outcomes in patients with intracranial tumors: a correlation study

Supplementary Table 1. Classification of postoperative complications based on the Clavien-Dindo classification

Postoperative Complications	Number (%)
Grade I	
Fever (T > 38.5 C)	22 (20.0%)
Electrolyte disorders	28 (25.5%)
Acid-Base disorders	8 (7.3%)
Incision infection	4 (3.6%)
Postoperative nausea and vomiting	23 (20.9%)
Grade II	
Acid-Base disorders	2 (1.8%)
Coagulation disorders	6 (5.5%)
Hypoproteinemia	18 (16.4%)
Incision infection	8 (7.3%)
Intracranial infections	4 (3.6%)
Pneumonia	17 (15.5%)
Urinary tract infection	1 (0.9%)
Sepsis	1 (0.9%)
Deep venous thrombosis	15 (13.6%)
Postoperative bleeding	5 (4.5%)
Seizure	14 (12.7%)
Delirium	18 (16.4%)
Cerebral infarction	4 (3.6%)
Grade III a	
Intracranial infections	7 (6.4%)
Grade III b	
Pneumonia	1 (0.9%)
Postoperative bleeding	2 (1.8%)
Grade IV a	
Single organ dysfunction	7 (6.4%)
Grade IV b	
Multiple organ dysfunction	1 (0.9%)
